# Self-Controllable Secure Location Sharing for Trajectory-Based Message Delivery on Cloud-Assisted VANETs

**DOI:** 10.3390/s18072112

**Published:** 2018-07-01

**Authors:** Youngho Park, Chul Sur, Si-Wan Noh, Kyung-Hyune Rhee

**Affiliations:** 1 Department of IT Convergence and Application Engineering, Pukyong National University, Busan 48513, Korea; pyhoya@pknu.ac.kr; 2 Department of Information Security, Busan University of Foreign Studies, Busan 46234, Korea; certless@gmail.com; 3 Interdisciplinary Program of Information Security, Graduate School, Pukyong National University, Busan 48513, Korea; nosiwan@pukyong.ac.kr

**Keywords:** VANETs, location sharing, authentication, privacy preservation, trajectory-based message delivery

## Abstract

In vehicular ad hoc networks, trajectory-based message delivery is a message forwarding strategy that utilizes the vehicle’s preferred driving routes information to deliver messages to the moving vehicles with the help of roadside units. For the purpose of supporting trajectory-based message delivery to a moving vehicle, the driving locations of the vehicle need to be shared with message senders. However, from a security perspective, vehicle users do not want their driving locations to be exposed to others except their desired senders for location privacy preservation. Therefore, in this paper, we propose a secure location-sharing system to allow a vehicle user (or driver) to share his/her driving trajectory information with roadside units authorized by the user. To design the proposed system, we put a central service manager which maintains vehicle trajectory data and acts as a broker between vehicles and roadside units to share the trajectory data on the cloud. Nevertheless, we make the trajectory data be hidden from not only unauthorized entities but also the service manager by taking advantage of a proxy re-encryption scheme. Hence, a vehicle can control that only the roadside units designated by the vehicle can access the trajectory data of the vehicle.

## 1. Introduction

For the last decade, vehicular ad hoc networks (VANETs) have attracted a great deal of attentions due to the development of vehicle-to-vehicle (V2V) and vehicle-to-infrastructure (V2I) communication technologies. It is a trend of modern vehicles to equip on-board unit (OBU) devices which allow vehicles to communicate with each other as well as roadside units (RSUs) [1]. As a result, up to date, various VANET applications using V2V and V2I communications have been presented to realize not only comfortable driving conditions but also valuable infotainment services on the road. Furthermore, recently, the concept of vehicular networking is extended to vehicular cloud computing by integrating vehicular communications with cloud computing to provide vehicles with pervasive services on the road [2,3,4].

One of the promising applications is a location-aware service [5] which provides vehicles with useful information for a certain geographic area of interest by taking advantage of vehicular cloud computing. Based on vehicular cloud computing technologies, RSUs deployed on the certain areas can collect and provide local-interesting information to vehicles such as traffic conditions and available facilities. However, it is challenging on the VANET how we can effectively deliver such service messages to vehicles which are continuously moving on the road. Due to the dynamic mobility and opportunistic connectivity in VANETs, the probability of successful hop-by-hop (among neighboring vehicles) message forwarding to a destination vehicle at a long distance is low and it would result in high message loss. As a solution to this challenge, trajectory-based message delivery with the help of RSUs in VANETs have been researched [6,7].

**Figure 1:**
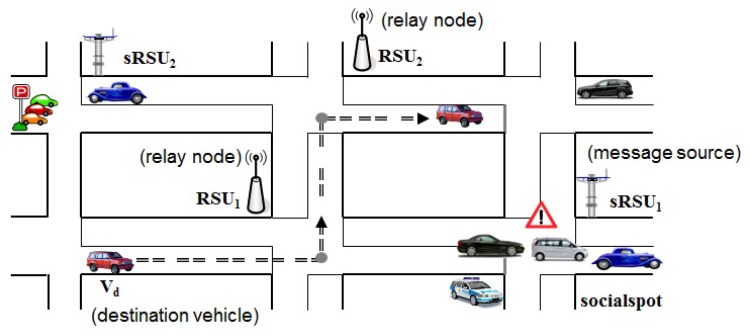
Service scenario of trajectory-based message delivery.

For example, let us consider a scenario as shown in Figure 1 in which the area around ${\mathrm{sRSU}}_{1}$ is an interested spot (named socialspot) of a vehicle $V_{d}$ and ${\mathrm{sRSU}}_{1}$ provides location-aware service around its local area to $V_{d}$. By using trajectory-based message delivery, it would be possible that ${\mathrm{sRSU}}_{1}$ can efficiently disseminate the service messages to $V_{d}$ through ${\mathrm{RSU}}_{1}$ and ${\mathrm{RSU}}_{2}$ acting as relay nodes along $V_{d}$’s trajectory assuming that ${\mathrm{sRSU}}_{1}$ knows the driving path of $V_{d}$.

On the other hand, driving route or location information of a vehicle is regarded as personal data of the driver, so location privacy is one of the critical security requirements for VANETs as well as identity privacy preservation. In the above trajectory-based message delivery environment, if a vehicle needs to share its driving route with message senders (sRSUs) deployed on some socialspots in which the vehicle is interested for location-aware service, how to share vehicle’s trajectory securely with the sRSUs in the system for privacy preservation is required. In other words, the system must be carefully designed not to expose the driving locations of a vehicle to other entities except the sRSUs designated by the vehicle.

### 1.1. Related Work

While various VANET applications have been emerging, studies on secure vehicular communications have also been widely performed [4,8,9,10,11,12,13,14,15,16]. One of the challenging issues is privacy-preserving vehicular communications for protecting location privacy of vehicles in VANETs. To prevent a global eavesdropping attacker from tracking a target vehicle, most privacy-preserving secure vehicular communications recommend using anonymous authentication schemes based on a group signature or pseudonymous credentials of vehicles when exchanging messages [17]. In addition to sender’s anonymity, to achieve message receiver’s location privacy preservation in VANETs, Lu et al. [18] and Lin et al. [19] proposed secure RSU-assisted message delivery protocols from a source vehicle to a destination vehicle, respectively. However, their work assumes that a receiver vehicle is stationary at a fixed location [18] and the location of a receiver is already known to senders beforehand [19].

For efficient message delivery from an RSU to moving vehicles in VANETS, Jeong et al. proposed an architecture for trajectory-based data delivery [7]. Their work was not focused on security mechanisms for protecting location privacy of vehicles. Instead, they just assume that a control center maintains vehicle trajectories and will not expose the trajectory data to others. In their system, we cannot help but rely on the control center that the center will carry out its role faithfully. However, from user’s viewpoint, the control center may be also a source of concern and users want to control who can access their trajectory data by themselves with a proper security mechanism.

With regard to secure location-sharing in mobile environments, the works in [20,21,22,23,24] proposed some methods to protect user’s privacy for location-sharing in mobile online social network services. They are mainly focused on a proximity application to find a nearby friend whose current location is within some distance. Some previous work did not consider preventing a service provider from accessing user’s locations data except the work of [20,24]. Even though a service provider is usually involved in location sharing services to distribute the location data of one user to other authorized users, the provider does not need to know the data content. Freudiger et al. [20] and Li et al. [24] proposed a system for protecting user location data from the provider by using data encryption, respectively. However, if a user wants to share his/her location data with multiple friends, the user has to generate multiple encrypted data for the same location data under a different key of each friend or needs interactions to establish a shared secret key with his friends. It burdens the user with computation and communication overheads proportional to the number of friends.

Dong et al. introduced a concept of secure location-sharing based on a proxy re-encryption scheme for mobile applications [25]. Proxy re-encryption is a cryptographic technique to allow a semi-trusted proxy to convert a ciphertext under one party’s public key (e.g., $V_{d}$ in our scenario) into a new ciphertext under another party’s public key (e.g., sRSU designated by $V_{d}$). While the proxy uses re-encryption keys to perform the conversion, the proxy cannot learn any information about the underlying plaintext. Dong et al. presented an idea that a user can efficiently share its location data with other users as shifting computational overheads for distributing encrypted location data to a proxy, and their idea motivated our work. However, if we adopt an ordinary public key based proxy re-encryption scheme, sRSU has to be involved in generating a re-encryption key to be used for converting a ciphertext by giving its public key certificate to $V_{d}$. Moreover, to revoke a re-encryption key of an unwanted sRSU and update re-encryption keys of other valid parties, $V_{d}$ must change its public key and obtain a new public key certificate. Such interactions are not always available in VANET environments due to the occasional connectivity. Therefore, we need to devise a more flexible and practical method to manage the keys for proxy re-encryption scheme in our VANET service scenario.

In our previous work [26], we presented a secure location sharing based on an id-based proxy re-encryption scheme as considering the non-interactive property of id-based public key cryptography. However, to revoke or update re-encryption keys in the previous work, this system needs to renew user’s identity functioning as a public key but it may be impractical to change the identity used in the system. As an alternative, in this paper, we employ a certificateless proxy re-encryption scheme which can provide more flexible key management to our self-controllable secure location-sharing system.

### 1.2. Our Contribution

Based on the above considerations, in this paper, we propose a secure driving location (trajectory) data sharing system for trajectory-based message delivery in VANETs which can prevent the shared location data on the cloud from being illegitimately exposed to others. We consider a location-aware service scenario in which a vehicle allows some socialspot RSUs designated by the vehicle to access its preferred driving trajectory data stored on the cloud. For secure vehicular communications, group signatures or pseudonymous credentials have been used for anonymous authentication so that the identity and location of a vehicle cannot be linked and tracked on VANETs. However, the main goal of our work is to securely share the trajectory data of a vehicle with only authorized entities from confidentiality view point and to control who can access the location data by vehicle itself. To achieve our goal, we present a system architecture for sharing the trajectory data of vehicles with the help of a service manager acting as a broker between vehicles and socialspot RSUs, then design a secure location-sharing and authenticated message delivery protocols by making use of a certificateless proxy re-encryption scheme and an id-based signature scheme with pseudonymous identities.

In our proposed system, a vehicle can upload driving trajectory data encrypted under its own public key to a semi-trusted service manager on the cloud. The uploaded trajectory data are coupled with re-encryption keys associated with the designated socialspot RSUs so that the manager re-encrypts vehicle’s trajectory data and distributes to the intended socialspot RSUs. Then, the socialspot RSUs can send service messages to the vehicle by way of some RSUs along the driving route of the vehicle. We make the vehicle revoke the access rights of unwanted socialspot RSUs by changing vehicle’s public key and re-encryption keys but the vehicle does not need to obtain a certificate for the new public key. Therefore, the proposed system can be self-controllable and more flexible.

The rest of this paper is organized as follows: We first present a system architecture and security considerations in Section 2 and cryptographic building blocks to design the proposed system in Section 3. Then, we describe our proposed secure location-sharing and authenticated message delivery protocols in Section 4. We discuss the security and performance of our protocol in Section 5, and finally conclude this paper in Section 6.

## 2. System Architecture

### 2.1. Architecture

**Figure 2:**
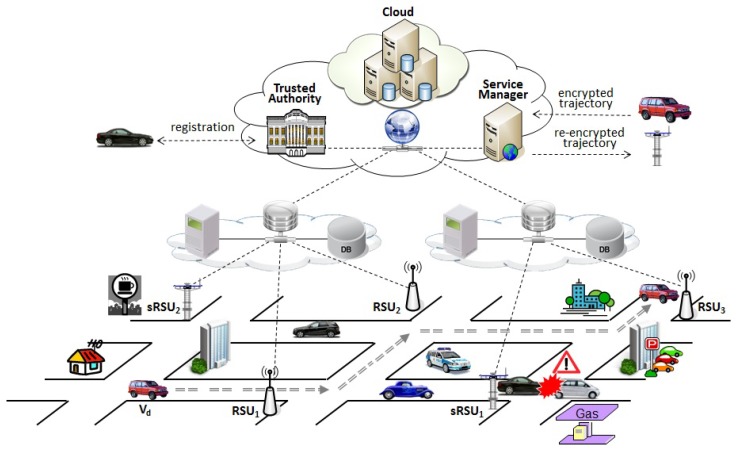
System Architecture.

To design a secure location-sharing system for trajectory-based message delivery on VANETs, we consider the system architecture shown in Figure 2 which consists of trusted authority (TA), service manager (SM), roadside units, and vehicles.

- TA is a fully trusted entity responsible for managing security parameters for the system and issues id-based key pairs to the registered RSUs and vehicles denoted as $R=\{RSU_{1},\ldots ,RSU_{m}\}$ and $V=\{V_{1},\ldots .,V_{n}\}$, respectively. TA also manages pseudonymous identities for the vehicles to guarantee anonymity of vehicles on VANET communications.
- SM is a manager which provides storage service on the cloud. To support secure trajectory data sharing, SM maintains encrypted trajectory data of vehicles and acts as a broker which handles to distribute re-encrypted trajectory data to RSUs authorized by the vehicle of the trajectory owner.
- RSUs are subordinated to the TA and sparsely deployed on the roads such as main intersections, and their geographical location information are available through the system. The roles of RSUs in R are divided into socialspot RSUs and relaying RSUs. Socialspot RSUs (sRSUs), denoted as $\mathrm{SR}=\{sRSU_{1},\ldots ,sRSU_{l}\}\subseteq R$, are deployed on the specific locations of interest. A set of RSUs establish a local cloud with dedicated servers so that they collect and provide location-aware information to vehicles by means of trajectory-based message delivery. On the other hand, a relaying node RSU equips storage for temporarily holding messages to support message forwarding to the destination vehicles passing by its coverage.
- Vehicles are equipped with OBU and GPS-based navigation system with digital maps. A registering vehicle $V_{d}\in V$ selects sRSUs among SR in which $V_{d}$ is interested and generates a re-encryption key for the selected sRSUs to share its driving trajectory data through the cloud storage under the control of SM. Whenever $V_{d}$ changes its preferred driving route, $V_{d}$ uploads its encrypted driving route data to SM.

Moreover, to clarify the proposed system, we also make the following assumptions.

- Public security parameters of TA are already known to all entities in the system.
- SM and socialspot RSUs are interconnected to each other through a secure and reliable channel.
- Locations and identities of RSUs are publicly available to the system so that vehicles can know which RSUs are deployed at which locations.

### 2.2. Threat Model and Security Considerations

In our system architecture, we consider two types of attack. One is an attack by a compromised SM. In other words, we assume that SM is a honest-but-curious entity that will follow the protocol but may try to extract vehicle’s driving trajectory data stored on the cloud for the purpose of collecting and profiling driver’s preferences. The other is an attack by an outside attacker which has no access privilege to the cloud but try to learn vehicle’s driving locations over VANET communications. However, when we assume that SM and RSUs are interconnected through a secure channel, it is hard for an outsider attacker to control the communications between SM and an RSU. In addition, we assume that all RSUs are trusted under the control of the TA so that SM is not allowed to collude with any RSU. Thus, it is assumed that TA can inspect all RSUs subordinated to it and the collusion or compromise of an RSU is detectable by the TA.

Under the threat model and assumptions, we consider the following security requirements to design a secure vehicle trajectory data sharing system and message delivery protocol to guarantee the location privacy of vehicles in VANETs.

- *Authorized access to trajectory data*: Access to driving trajectory data of a vehicle on the cloud must be restricted to only the RSUs authorized by the owner vehicle of the trajectory data. Even though vehicle’s trajectory data are managed under the control of SM, driving trajectory data must be hidden from SM as well as unauthorized entities.
- *Self-control*: When a vehicle uploads its trajectory data to SM, it should be possible for the vehicle to control that which RSUs can or cannot access its trajectory data by the vehicle itself.
- *Authenticated communications*: For secure message delivery on VANETs, vehicles and RSUs involved in message delivery must be authenticated to each others. In message forwarding, a relaying RSU must authenticate a vehicle to check if the vehicle is a valid destination specified in the message. Besides, a vehicle must be convinced that the received message originated from a valid source claimed in the message.
- *Avoiding location tracking*: While a vehicle connects to RSUs on its driving routes to receive messages, driving trajectory of the vehicle must not be tracked by an outside attacker on VANETs. That is, it must be hard for an outside attacker to learn that a vehicle has moved from and to which of RSUs by overhearing vehicle-to-RSU communications.

## 3. Preliminaries

Before presenting our proposed system, in this section, we briefly outline the properties of a bilinear map and introduce some cryptographic schemes using a bilinear map which serve as the basis of the proposed system. More specifically, we design the proposed system using certificateless proxy re-encryption and id-based signature schemes with pseudonymous identities of vehicles.

### 3.1. Bilinear Map

Let $G_{1}$ and $G_{2}$ be two cyclic groups of the same prime order *q*. There is a bilinear map $e:G_{1}\times G_{1}\to G_{2}$ satisfying the following properties.

- Bilinear: $\hat{e}\left(g^{a},g^{b}\right)=\hat{e}{\left(g,g\right)}^{ab}$, for all $a,b\in Z_{q}^{*}$ and $g\in G_{1}$.
- Non-degenerate: If *g* is a generator of $G_{1}$, then $\hat{e}\left(g,g\right)$ is a generator of $G_{2}$.
- Computable: $\hat{e}\left(g,h\right)$ is efficiently computable for any $g,h\in G_{1}$.

### 3.2. Cryptographic Building Blocks

As our building blocks, we adopt a certificateless proxy re-encryption (CL-PRE) scheme [27] and an id-based signature (ID-SIG) scheme [28] described in the followings.

- *Setup*(): A private key generator chooses bilinear map groups $\left(G_{1},G_{2}\right)$ of the same prime order *q*, random generators $g,h\in G_{1}$, and hash functions $H_{1}\;:\;{\left\{0,1\right\}}^{*}\;\to G_{1}$, $H_{2}:{\left\{0,1\right\}}^{*}\to \;Z_{q}^{*}$, $H_{3}:G_{2}\times G_{2}\to {\left\{0,1\right\}}^{n+\kappa_{0}}$. It also chooses a random $s\in Z_{q}^{*}$ as a master secret then computes $g_{1}=g^{s}$, $g_{2}=\hat{e}\left(g,g\right)$, $g_{3}=\hat{e}\left(g,h\right)$. Public system parameters are set as $params:=\;\langle G_{1},G_{2},q,\hat{e},g,h,g_{1},g_{2},g_{3},H_{1},H_{2},H_{3}\rangle$.
- *idKeyGen*(*s*, id): Given an identity id, this algorithm outputs an id-based private key $d_{id}\;=\;g^{1/(s+H_{2}\left(id\right))}$ under the master secret key *s* of a private key generator.
- *SetPrvKey*($d_{id}$): Given a $d_{id}$ generated by *idKeyGen* for an id, this algorithm chooses random values $a,b\in Z_{q}^{*}$ and sets $sk_{id}=\left(d_{id},a,b\right)$ as a private key for CL-PRE for a user of the id.
- *SetPubKey*($sk_{id}$): This algorithm returns a public key $pk_{id}=\left(g_{3}^{a},g^{b}\right)$ corresponding to $sk_{id}$ for CL-PRE.
- *clEnc*(*m*, id, $pk_{id}$): Certificateless encryption algorithm generates a ciphertext for a given message *m* under the id and $pk_{id}$ as follows:
  1. Choose a random $\alpha \in {\left\{0,1\right\}}^{\kappa_{0}}$ for a security parameter $\kappa_{0}$.
  2. Compute $\beta =H_{2}(m|\alpha |id|pk_{id})$.
  3. Compute $c_{1}={\left(g^{H_{2}\left(id\right)}\cdot g_{1}\right)}^{\beta }$, $c_{2}=h^{\beta }$, $c_{3}=\left(m|\alpha \right)⊕H_{3}\left(g_{2}^{\beta }|{\left(g_{3}^{a}\right)}^{\beta }\right)$, and $c_{4}=u^{\beta }$ where $u=H_{1}(id|pk_{id}|c_{1}|c_{2}\left|c_{3}\right)$.
  4. Output the ciphertext $C=(c_{1},c_{2},c_{3},c_{4})$.
- *clReKeyGen*($sk_{id_{i}}$, $id_{i}$, $id_{j}$, $pk_{id_{i}}$, $pk_{id_{j}}$): Re-encryption key generation algorithm returns a proxy re-encryption key $rk_{id_{i}\to id_{j}}$ as follows:
  1. Choose a random $\gamma \in Z_{q}^{*}$ and compute $\mu =H_{2}(g_{2}^{\gamma }|id_{i}|pk_{id_{i}}|id_{j}\left|pk_{id_{j}}\right)$.
  2. Compute $rk^{\left(1\right)}=g^{\mu /(s+H_{2}\left(id_{i}\right))}$, $rk^{\left(2\right)}={\left(g^{H_{2}\left(id_{j}\right)}\cdot g_{1}\right)}^{\gamma }$, and $rk^{\left(3\right)}={\left(g^{b_{j}}\right)}^{a_{i}}$ where $a_{i}\in sk_{id_{i}}$ and $g^{b_{j}}\in pk_{id_{j}}$, respectively.
  3. Output the re-encryption key $rk_{id_{i}\to id_{j}}=\left(rk^{\left(1\right)},rk^{\left(2\right)},rk^{\left(3\right)}\right)$.
- *clReEnc*($id_{i}$, $pk_{id_{i}}$, *C*, $rk_{i\to j}$): Given a ciphertext *C* under the identity $id_{i}$ and public key $pk_{id_{i}}$, this algorithm outputs a re-encrypted ciphertext $C_{j}$ delegated to $id_{j}$ under the $rk_{id_{i}\to id_{j}}$ as follows:
  1. Parse *C* as $C=(c_{1},c_{2},c_{3},c_{4})$
  2. Compute $u=H_{1}(id_{i}|pk_{id_{i}}|c_{1}|c_{2}\left|c_{3}\right)$ and $y_{i}=H_{2}\left(id_{i}\right)$.
  3. If $\hat{e}\left(c_{1},u\right)\overset{?}{=}\hat{e}\left(\left(g^{y_{i}}\cdot g_{1}\right),c_{4}\right)$ and $\hat{e}\left(c_{2},u\right)\overset{?}{=}\hat{e}\left(h,c_{4}\right)$ holds,
  4. Set the re-encrypted ciphertext $C_{j}=\left(c_{1}^{\prime },c_{2}^{\prime },c_{3}^{\prime },c_{4}^{\prime },id_{i},pk_{id_{i}}\right)$, where $c_{1}^{\prime }=\hat{e}\left(c_{1},rk^{\left(1\right)}\right)$, $c_{2}^{\prime }\;=\;rk^{\left(2\right)}$, $c_{3}^{\prime }=\hat{e}\left(c_{2},rk^{\left(3\right)}\right)$, and $c_{4}^{\prime }=c_{3}$.
- *clReDec*($sk_{id_{j}}$, $C_{j}$): Given a re-encrypted ciphertext $C_{j}$ delegated to $id_{j}$ from $id_{i}$, decryption algorithm outputs the message *m* as follows:
  1. Parse $C_{j}$ as $C_{j}=\left(c_{1}^{\prime },c_{2}^{\prime },c_{3}^{\prime },c_{4}^{\prime },id_{i},pk_{id_{i}}\right)$.
  2. Compute $\rho =\hat{e}\left(c_{2}^{\prime },d_{id_{j}}\right)$ where $d_{id_{j}}\in sk_{id_{j}}$, and $\mu =H_{2}(\rho |id_{i}|pk_{id_{i}}|id_{j}\left|pk_{id_{j}}\right)$.
  3. Compute $\left(m|\alpha \right)=c_{4}^{\prime }⊕H_{3}\left({c_{1}^{\prime }}^{1/\mu }|{c_{3}^{\prime }}^{1/b_{j}}\right)$.
  4. Compute $\beta =H_{2}(m|\alpha |id_{i}\left|pk_{id_{i}}\right)$.
  5. If $c_{1}^{\prime }\overset{?}{=}g_{2}^{\mu \beta }$ and $c_{3}^{\prime }\overset{?}{=}{\left(g_{3}^{a_{i}}\right)}^{\beta b_{j}}$ holds, return *m*. Otherwise output ⊥.
- *idSig*($d_{id}$, *m*): On input an id-based secret key $d_{id}$ and a message *m*, this algorithm computes a signature *S* for the *m* as follows:
  1. Pick a random $x\in Z_{q}^{*}$ and compute $\theta =g_{2}^{x}$.
  2. Set the signature $S=(\sigma_{1},\sigma_{2})$, where $\sigma_{1}=H_{2}\left(m,\theta \right)$ and $\sigma_{2}=d_{id}^{x+\sigma_{1}}$.
- *idVrf*(id, *m*, *S*): ID-based signature verification algorithm accepts the message *m* if $\sigma_{1}\overset{?}{=}H_{2}\left(m,A\right)$ holds, where $A=\hat{e}\left(\sigma_{2},g^{H_{2}\left(id\right)}g_{1}\right)\cdot g_{2}^{-\sigma_{1}}$.

## 4. Proposed System

In this section, we describe the proposed secure trajectory data sharing system for supporting trajectory-based message delivery protocol on VANETs by making use of cryptographic techniques presented in Section 3. The proposed system consists of *setup*, *enrolment*, *trajectory sharing*, and *message delivery* phases. Table 1 shows the notations used to describe the proposed protocol.

### 4.1. Setup

TA first picks a random $s\in Z_{q}^{*}$ as its master secret and runs *Setup*() algorithm to generate public system parameters $params:=\langle G_{1},G_{2},q,\hat{e},g,h,g_{1},g_{2},g_{3},H_{1},H_{2},H_{3}\rangle$. TA also issues id-based secret keys to RSUs and vehicles registered to the system. Note, at this phase, we assume that id-based secret keys can be issued to RSUs and vehicles through an out-of-band secure channel before they participate in VANETs. Let $R=\{RSU_{1},\ldots ,RSU_{m}\}$ be the RSUs subordinated by the TA and $V=\{V_{1},\ldots ,V_{n}\}$ be the registering vehicles. For each $RSU_{j}\in R$, TA generates $RSU_{j}$’s id-based secret key $rsk_{j}\leftarrow$ *idKeyGen*(*s*, $RSU_{j}$) and preloads $rsk_{j}$ to each $RSU_{j}$ securely. Then, $RSU_{j}$ sets its private key as $sk_{RSU_{j}}=\left(rsk_{j},a_{j},b_{j}\right)\leftarrow$ *SetPrvKey*($rsk_{j}$) and public key $pk_{RSU_{j}}=\left(g_{3}^{a_{j}},g^{b_{j}}\right)\leftarrow$ *SetPubKey*($sk_{RSU_{j}}$) for CL-PRE.

On the other hand, for a vehicle $V_{d}\in V$, TA chooses w+1 pseudonymous identities $PID_{d}\;=\;\left\{pid_{di}|0\leq i\leq w\right\}$ for $V_{d}$ after checking the eligibility of $V_{d}$. TA issues $V_{d}$’s id-based secret keys $vsk_{di}\leftarrow$ *idKeyGen*(*s*, $pid_{di}$) for each $pid_{di}\in PID_{d}$. In our system, $pid_{d0}$ is used for re-encryption procedure while $\left\{pid_{d1},\ldots ,pid_{dw}\right\}$ are used for VANET communications. When obtaining id-based keys for the pseudonyms, $V_{d}$ sets its private key and public key for proxy re-encryption procedure as $sk_{V_{d}}=\left(vsk_{d0},a_{d},b_{d}\right)\leftarrow$ *SetPrvKey*($vsk_{d0}$) and public key $pk_{V_{d}}=\left(g_{3}^{a_{d}},g^{b_{d}}\right)\leftarrow$ *SetPubKey*($sk_{V_{d}}$) for CL-PRE. Then, each $V_{d}$ and each $RSU_{j}$, respectively, store $\left(pid_{d0},pk_{V_{d}}\right)$ and $\left(RSU_{j},pk_{RSU_{j}}\right)$ to the cloud storage so as for them to retrieve the public key of each others.

### 4.2. Enrolment

For a vehicle to receive location-aware service messages by means of trajectory-based message delivery, $V_{d}$ configures desired socialspots in which $V_{d}$ is interested for receiving the service messages and selects sRSUs installed at the socialspots denoted as $SR_{d}=\left\{sRSU_{j}|1\leq j\leq k\right\}\subseteq \mathrm{SR}$. Then, to allow each $sRSU_{j}\in SR_{d}$ to access $V_{d}$’s trajectory data under the control of SM using a proxy re-encryption scheme, $V_{d}$ generates the re-encryption key $rk_{V_{d}\to sRSU_{j}}$ for each $sRSU_{j}$ as follows:

1. Retrieve $sRSU_{j}$’s public key $pk_{sRSU_{j}}$ from the cloud storage.
2. Set a re-encryption key as $rk_{V_{d}\to sRSU_{j}}\leftarrow$ *clReKeyGen*($sk_{V_{d}}$, $pid_{d0}$, $sRSU_{j}$, $pk_{V_{d}}$, $pk_{sRSU_{j}}$).
3. Compose a message $RSM_{d}=\{pid_{d0},pk_{V_{d}},SR_{d}$, $RK_{d}\}$, where $RK_{d}=\left\{rk_{V_{d}\to sRSU_{j}}\;|\;sRSU_{j}\in SR_{d}\right\}$.

$V_{d}$ entrusts $RSM_{d}$, consisting of sRSUs list and re-encryption keys, to SM so as for SM to re-encrypt $V_{d}$’s trajectory data and delegate decryption right to each $sRSU_{j}$ when $V_{d}$ uploads its encrypted trajectory data to SM.

### 4.3. Trajectory Sharing on the Cloud

Let $T_{d}=\left\{loc_{1}\to \ldots \to loc_{t}\right\}$ be the driving trajectory of a vehicle $V_{d}$ consisting of some specific locations such as main roads or intersections on $V_{d}$’s preference driving routes. When $V_{d}$ participates in VANETs, $V_{d}$ will expects to receive service messages provided by the socialspot RSUs ($SR_{d}$) of $V_{d}$’s interest through some contact point RSUs deployed on $V_{d}$’s driving trajectory $T_{d}$. To securely share $V_{d}$’s trajectory data and pseudonyms used for message delivery in VANETs with the socialspot RSUs in $SR_{d}$ through the cloud storage, $V_{d}$ uploads encrypted trajectory data and pseudonyms to SM as follows:

1. Choose a pseudonym $pid_{di}\in PID_{d}$ to be used for connecting to a contact point $RSU_{i}$.
2. Compose a pseudonym-location pair message $trj_{d}=\left\{\left(pid_{di},loc_{i}\right)|1\leq i\leq t\right\}$.
3. Generate a ciphertext *C* for $trj_{d}$ as C← *clEnc*($trj_{d}$, $pid_{d0}$, $pk_{V_{d}}$) under $V_{d}$’s own public key.
4. Upload $TM_{d}=\left\{pid_{d0},C,ts,S_{d}\right\}$ to SM, where $S_{d}\leftarrow$ *idSig*($vsk_{d0}$, $pid_{d0}\left|C\right|ts$) is $V_{d}$’s signature under the id-based secret key $vsk_{d0}$ of $pid_{d0}$.

Once $V_{d}$ uploads its trajectory sharing message $TM_{d}$ to the cloud storage, SM controls to transform and provide $V_{d}$’s encrypted trajectory to the sRSUs in $SR_{d}$ specified by $V_{d}$ as follows:

1. Parse $TM_{d}$ as $\left\{pid_{d0},C,ts,S_{d}\right\}$ and verify the signature $S_{d}$ as *idVrf*($pid_{d0}$, $pid_{d0}\left|C\right|ts$, $S_{d}$) under the given $pid_{d0}$.
2. Retrieve $RSM_{d}=\left\{pid_{d0},pk_{V_{d}},SR_{d},RK_{d}\right\}$ for the given $pid_{d0}$ from SM’s storage.
3. For each $sRSU_{j}\in SR_{d}$, extract $rk_{V_{d}\to sRSU_{j}}\in RK_{d}$ and transform the ciphertext *C* to $C_{j}^{\prime }$ as $C_{j}^{\prime }\leftarrow$ *clReEnc* ($pid_{d0}$, $pk_{V_{d}}$, *C*, $rk_{V_{d}\to sRSU_{j}}$).
4. Store $\left\{C_{j}^{\prime },ts\right\}$ to $sRSU_{j}$’s directory on the cloud.

While SM maintains vehicles encrypted trajectory information, each socialspot RSU, $sRSU_{j}$, periodically access its directory to get the updated trajectory of $V_{d}$ as follows:

1. Downloads $\left\{C_{j}^{\prime },ts\right\}$ from its directory on the cloud.
2. Decrypt $C_{j}^{\prime }$ to get $trj_{d}$ as $trj_{d}=\left\{\left(pid_{di},loc_{i}\right)|1\leq i\leq t\right\}\leftarrow$ *clReDec*($sk_{sRSU_{j}}$, $C_{j}^{\prime }$).
3. Add $\left(pid_{di},loc_{i}\right)$ pairs to the vehicle list VList.

### 4.4. Trajectory-based Message Delivery

**Figure 3:**
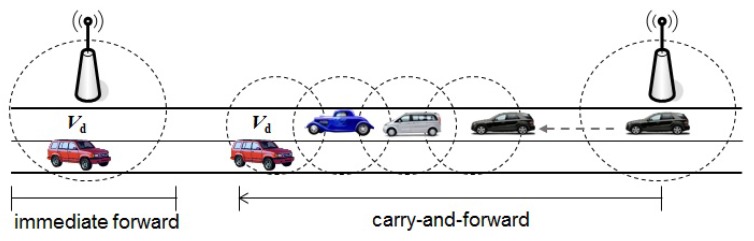
Message forwarding from an RSU to a destination vehicle.

Suppose that $sRSU_{j}$ collects and provides location-aware service information, such as traffic condition, gas station, and so on. Once $trj_{d}=\left\{\left(pid_{di},loc_{i}\right)\right\}$ of $V_{d}$ is loaded, $sRSU_{j}$ can disseminate location-aware service messages for $V_{d}$ through an $RSU_{i}$ deployed on around $loc_{i}$. Message delivery from $sRSU_{j}$ to $V_{d}$ can be subdivided into three phases: (1) message distribution of $sRSU_{j}$ to $RSU_{i}$; (2) immediate message forwarding by $RSU_{i}$ to $V_{d}$ if $V_{d}$ is within $RSU_{i}$’s transmission coverage; and (3) message carry-and-forwarding by other vehicles to $V_{d}$ if $V_{d}$ is out of $RSU_{i}$’s transmission coverage, as shown in the Figure 3.

#### 4.4.1. Message Distribution to RSUs

Let msg be a content of location-aware service provided by $sRSU_{j}$. In this phase, $sRSU_{j}$ compose a message package $M_{jd}$ for $V_{d}$ and distributes $M_{jd}$ to $V_{d}$’s contact point $RSU_{i}$ as follows:

1. Extract $pid_{di}$ corresponding to $loc_{i}$ from VList.
2. Set a message $M_{jd}=\left\{pid_{di},sRSU_{j},msg,ts^{\prime },S_{j},ttl\right\}$ where $S_{j}\leftarrow idSig(rsk_{j},pid_{di}|sRSU_{j}|msg|ts^{\prime })$ is $sRSU_{j}$’s signature and ttl is the message lifetime.
3. Distribute $M_{jd}$ to $RSU_{i}$ nearby $loc_{i}$.

When we assume that RSUs are inter-connected, a message $M_{jd}$ can be easily distributed to other RSUs. During the message delivery, a message bundle has a certain lifetime specified as ttl so that an expired message bundle is discarded. This is for RSUs or carrying vehicles to avoid consuming their storage excessively for a long time even if a target vehicle $V_{d}$ is not met on the roads.

#### 4.4.2. Immediate Message Forwarding

If a message is distributed to contact point RSUs, each RSU stores the received messages and forwards them when a target vehicles for the message passes by RSU’s coverage before ttl is expired. Suppose that a vehicle $V_{d}$ enters $RSU_{i}$’s coverage and $M_{jd}$ sent from $sRSU_{j}$ for $V_{d}$ is kept by $RSU_{i}$. The followings describe message forwarding protocol from $RSU_{i}$ to $V_{d}$.

1. $RSU_{i}$ periodically broadcasts beacon message containing $pid_{di}$ specified as a destination of $M_{jd}$.
2. If $pid_{di}$ is found in the beacon message, $V_{d}$ sends a message request $\left\{req,pid_{di},S_{d}^{\prime }\right\}$ to $RSU_{i}$, where req is a metadata for message requesting and $S_{d}^{\prime }\leftarrow$ *idSig*($vsk_{di}$, $req|pid_{di}$).
3. Upon receiving the request message, $RSU_{i}$ authenticates $V_{d}$ by verifying $S_{d}^{\prime }$ as *idVrf*($pid_{di}$, $req|pid_{di}$, $S_{d}^{\prime }$). If it holds, $RSU_{i}$ sends $M_{jd}$ to $V_{d}$.

When $M_{jd}$ is downloaded, $V_{d}$ checks the authenticity of the received message msg to be convinced whether the message actually originated from $V_{d}$’s desired $sRSU_{j}$ as follows:

1. Parse $M_{jd}=\left\{pid_{di},sRSU_{j},msg,ts^{\prime },S_{j},ttl\right\}$.
2. Verify the signature $S_{j}$ as $idVrf(sRSU_{j},pid_{di}|sRSU_{j}|msg|ts^{\prime },S_{j})$; and, if it holds,
3. Accept msg as a valid message from $sRSU_{j}$.

#### 4.4.3. Message Carry-and-Forwarding

If we consider that a target vehicle drives beyond contact point RSU’s transmission coverage, another strategy to deliver a message is to rely on VANET routing by means of carry-and-forwarding among neighboring vehicles on the road. For instance, to deliver a message $M_{jd}$ on VANET, $RSU_{i}$ requests and finds a volunteer vehicle which willingly joins carrying and forwarding the message. Suppose that a vehicle $V_{c}$ within $RSU_{i}$’s range is a volunteer vehicle. For $RSU_{i}$’s request, $V_{c}$ responds acceptance message $\left\{acc,pid_{ci},S_{c}\right\}$ to $RSU_{i}$ to obtain $M_{jd}$, where acc is a metadata and $S_{c}\leftarrow$ *idSig*($pid_{ci}$, $acc|pid_{ci}$). $RSU_{i}$ authenticates $V_{c}$ by verifying the signature $S_{c}$ under $pid_{ci}$, if it holds, provides $V_{c}$ with $M_{jd}$.

Once $M_{jd}$ is stored in $V_{c}$’s storage, $V_{c}$ carries $M_{jd}$ by itself until $V_{c}$ runs into the target vehicle $V_{d}$ of $pid_{di}$ on the road before ttl is expired, or $V_{c}$ forwards $M_{jd}$ to a next-hop vehicle in accordance with VANET routing protocol. For the former case, if $V_{c}$ detects $V_{d}$ on driving, $V_{c}$ sends notification message to $V_{d}$ to inform that $V_{c}$ has a message $M_{dj}$ for $V_{d}$. Then, $V_{d}$ requests the message to $V_{c}$ in authenticated manner as follows:

1. $V_{d}$ requests the message to $V_{c}$ by sending $\left\{req,pid_{di},S_{d}^{\prime }\right\}$.
2. $V_{c}$ verifies the signature $S_{d}^{\prime }$ and forwards $M_{jd}$ attached with its signature as $\{res,pid_{ci},M_{jd},\;S_{c}\leftarrow$ *idSig*($vsk_{ci}$, $res|pid_{ci}|M_{jd}$)}, where res is a metadata for the response.
3. $V_{d}$ first verifies $V_{c}$’s signature $S_{c}$ and extracts $M_{jd}$. Then, $V_{d}$ checks if $M_{jd}$ actually originated from $sRSU_{j}$ by checking $sRSU_{j}$’s signature $S_{j}$ in $M_{jd}$ as described in step 2) of immediate forwarding.

On the other hand, if a message is delivered by hop-by-hop forwarding, each intermediary vehicle involved in VANET routing protocol must append its signature to the forwarded message. For instance, suppose that $hop=\{V_{1}\to V_{2}\to \ldots \to V_{l}\}$ is a set of vehicles involved in message forwarding to the destination vehicle $V_{d}$ (Note, how to establish a route and forward a message to the destination node is beyond our scope. We can assume the existing VANET routing protocols such as [6,29]). The message arriving to $V_{d}$ consists of $\left\{fwd,M_{jd},{\left\{pid_{hi}|S_{h}\right\}}_{V_{h}\in hop}\right\}$ where fwd is a metadata for message forwarding and $S_{h}$ is a signature of each $V_{h}$. The last hop vehicle can forward $M_{jd}$ to $V_{d}$ as described in the above. By verifying each signature $S_{h}$ under $pid_{hi}$, $V_{d}$ can be convinced that the received message $M_{jd}$ was forwarded via authenticated vehicles.

### 4.5. Trajectory Update and Sharing Revocation

After vehicles initially upload their trajectory data to SM, they can update their trajectory data whenever they want to change preference driving routes. Remind, in trajectory sharing phase in Section 4.3, $V_{d}$’s trajectory $T_{d}$ is uploaded to the cloud in the encrypted form of *C* in $trj_{d}$, and then *C* is transformed to a re-encrypted ciphertext $C_{j}$ by the SM to be shared with an $sRSU_{j}\in SR_{d}$. Suppose that $V_{d}$’s trajectory $T_{d}$ is changed to different driving locations $T_{d}^{\prime }=\left\{loc_{1}^{\prime }\to \ldots \to loc_{t}^{\prime }\right\}$ but $V_{d}$ still wants to share the changed trajectory with the current sRSUs in $SR_{d}$. In this case, the operation required to $V_{d}$ is just to generate a ciphertext $C^{\prime }$ for the changed trajectory $T_{d}^{\prime }$ in accordance with the procedures of Section 4.3, and update $TM_{d}$ to include $C^{\prime }$ on the cloud storage. Then, SM can transform $C^{\prime }$ to $C_{j}^{\prime }$ for each $sRSU_{j}$ using the existing re-encryption keys $RK_{d}$ in $RSM_{d}$.

Sometimes, however, a vehicle will not wish to share the updated trajectory data with some sRSUs any more if the vehicle changes its interested socialspots. That is, a vehicle needs to revoke unwilling trajectory sharing. Suppose that $V_{d}$ does not want to share updated trajectory data with an $sRSU_{j}$. In the proposed system, $V_{d}$ can revoke trajectory sharing with $sRSU_{j}$ by updating $V_{d}$’s private and public key pair and re-encryption keys for sRSUs except the $sRSU_{j}$ as follows:

1. Renew its private key as $sk_{V_{d}}\;=\;\left(vsk_{d0},a_{d}^{\prime },b_{d}^{\prime }\right)\leftarrow$ *SetPrvKey*($vsk_{d0}$) and public key as $pk_{V_{d}}\;=\;\left(g_{3}^{a_{d}^{\prime }},g^{b_{d}^{\prime }}\right)\leftarrow$ *SetPubKey*($sk_{V_{d}}$) by choosing new random values $a_{d}^{\prime },b_{d}^{\prime }\in Z_{q}^{*}$.
2. Change the socialspot RSUs list as $SR_{d}$ to $SR_{d}^{\prime }$ by adding new sRSUs and deleting revoked sRSUs.
3. Set $RK_{d}^{\prime }=\left\{rk_{V_{d}\to sRSU_{i}}\;|\;sRSU_{i}\in SR_{d}^{\prime }\right\}$ by running $rk_{V_{d}\to sRSU_{i}}\leftarrow$ *clReKeyGen*($sk_{V_{d}}$, $pid_{d0}$, $sRSU_{i}$, $pk_{V_{d}}$, $pk_{sRSU_{i}}$) for each $sRSU_{i}$.
4. Replace the existing $RSM_{d}$ uploaded in enrolment phase of Section 4.2 with the updated $RSM_{d}^{\prime }$ including $RK_{d}^{\prime }$ and $SR_{d}^{\prime }$.

## 5. Analysis

### 5.1. Security

#### 5.1.1. Authorized Access to Trajectory Data

Since the locations of a vehicle can be regarded as personal information of a driver, driving trajectory data maintained on the cloud must not be exposed to not only outsiders but also SM illegally in the system. In our proposed system, a trajectory data $trj_{d}$ consisting of pseudonym-location pairs of a vehicle $V_{d}$ is maintained by SM in the encrypted form under $V_{d}$’s public key $pk_{V_{d}}$ as C← *clEnc*($trj_{d}$, $pid_{d0}$, $pk_{V_{d}}$). Essentially, nobody can gain $V_{d}$’s trajectory data as trying to decrypt *C* without knowing $V_{d}$’s private key $sk_{V_{d}}$ corresponding to $pk_{V_{d}}$. On the other hand, some sRSUs of $V_{d}$’s interested socialspots specified in $SR_{d}$ need to know $V_{d}$’s driving trajectories for disseminating service messages through contact point RSUs along $V_{d}$’s driving routes. $V_{d}$ can allow an $sRSU_{j}\in SR_{d}$ to get its trajectory data by giving a re-encryption key $rk_{V_{d}\to sRSU_{j}}\leftarrow$ *clReKeyGen*($sk_{V_{d}}$, $pid_{d0}$, $sRSU_{j}$, $pk_{V_{d}}$, $pk_{sRSU_{j}}$) to SM instead of entrusting plaintext trajectory data. The encrypted trajectory data *C* are then re-encrypted to $C_{j}^{\prime }\leftarrow$ *clReEnc* ($pid_{d0}$, $pk_{V_{d}}$, *C*, $rk_{V_{d}\to sRSU_{j}}$) for $sRSU_{j}$ under the key $rk_{V_{d}\to sRSU_{j}}$ by SM. Hence, only a valid $sRSU_{j}$ that possesses a private key $sk_{sRSU_{j}}$ corresponding to $pk_{sRSU_{j}}$ involved in $rk_{V_{d}\to sRSU_{j}}$ can get $trj_{d}$ by decrypting $C_{j}$.

In addition to an outside attacker and unauthorized RSUs, another concern is the security threat of SM to the trajectory data managed on the cloud since a semi-honest SM may be curious to know vehicle’s driving locations for the purpose of collecting and profiling driver’s preferences. At this phase, even though the encrypted trajectory data and re-encryption keys are given to SM, it is hard for SM to deduce the private key of $V_{d}$ or $sRSU_{j}$ for the purpose of recovering the trajectory data $trj_{d}$ from *C* or $C_{j}$ if we assume the security properties of the underlying proxy re-encryption scheme [27]. Therefore, neither SM nor unauthorized RSUs can access vehicle’s trajectory data on the cloud unless the vehicle authorizes decryption rights for the encrypted trajectory data under re-encryption keys.

#### 5.1.2. Self-Controllable Trajectory Sharing

In our proposed system, even though vehicle’s shared trajectory data are maintained and transferred by SM, it is the owner vehicle of the trajectory data that can decide what RSUs can access its trajectory data on the cloud. As mentioned before, distribution of the trajectory data of $V_{d}$ is performed by SM according to socialspot list $SR_{d}$ and re-encryption keys $RK_{d}=\left\{rk_{V_{d}\to sRSU_{j}}|sRSU_{j}\in SR_{d}\right\}$ generated by $V_{d}$ in the enrolment phase. If $V_{d}$ does not want an $sRSU_{j}$ existing in $SR_{d}$ to access its trajectory data any more, $V_{d}$ can revoke $sRSU_{j}$’s decryption right by generating new re-encryption keys for sRSUs in $SR_{d}$ except $sRSU_{j}$. Once $V_{d}$ uploads updated $SR_{d}^{\prime }\setminus \left\{sRSU_{j}\right\}$ and $RK_{d}^{\prime }$ for each sRSU in $SR_{d}^{\prime }$, SM will exclude $sRSU_{j}$ and not provide $sRSU_{j}$ with the re-encrypted trajectory data of $V_{d}$. In our revocation procedure of Section 4.5, $V_{d}$ can generate re-encryption keys without involvement of SM and sRSUs, and it does not require renewing public keys of sRSUs due to the functionality of certificateless proxy re-encryption scheme. Therefore, our proposed system can provide a flexible and self-controllable trajectory data sharing mechanism.

#### 5.1.3. Authenticated Vehicular Communications

To receive a service message in the form of $M=\{pid_{di},sRSU_{j},msg,ts^{\prime },S_{j},ttl\}$ served by sRSUs on VANETs, $V_{d}$ must be authenticated to a contact point $RSU_{i}$ or carrier vehicle $V_{c}$ as $V_{d}$ is the valid destination vehicle of $pid_{di}$ specified in the message *M*. In message forwarding phase of Section 4.4, when $V_{d}$ requests a message *M* kept by a contact point $RSU_{i}$ or a carrying vehicle $V_{c}$, $V_{d}$ must present its id-based signature $S_{d}^{\prime }\leftarrow$ *idSig*($vsk_{di}$, $req|pid_{di}$) which is in turn verified under the given $pid_{di}$ of the message *M*. If we assume the security of an id-based signature scheme, any vehicle which does not know the private key corresponding to $pid_{di}$ except $V_{d}$ cannot forge the signature nor impersonate $V_{d}$. Therefore, only the valid vehicle $V_{d}$ authenticated under the $pid_{di}$ can receive the message *M*.

In addition, when $V_{d}$ receives a message *M*, $V_{d}$ also authenticates the message sender sRSU by verifying the attached id-based signature $S_{j}$ under the sRUS’s identity $sRSU_{j}$ specified in *M*. That is, $V_{d}$ can be convinced that the message *M* was sent from the $sRSU_{j}$, in which $V_{d}$ is interested, if the signature $S_{j}$ is verified as valid under the id of $sRSU_{j}$.

#### 5.1.4. Avoiding Location Tracking

Due to the access control to the trajectory data, we can prevent an outside attacker as well as any unauthorized entity from learning vehicle’s driving trajectory stored on the cloud. On the other hand, to prevent an outside attacker from tracking driving path of a vehicle by eavesdropping on the vehicle-to-RSU communications, it must be difficult for an outside attacker to guess that the observed vehicle at different RSU’s coverage is the same vehicle while a vehicle connects to contact point RSUs for receiving a message over VANET on its driving. In our proposed system, a vehicle $V_{d}$ has a set of pseudonyms $\left\{pid_{d1},..,pid_{dw}\right\}$, which can be independently generated random values, and it is recommended to use a different pseudonym for identification and authentication whenever $V_{d}$ connects to a different contact point $RSU_{i}$ deployed at the location of $loc_{i}$ along $V_{d}$’s driving path. Therefore, we can make it hard for an outside attacker to track moving locations of a vehicle if any two pseudonyms $pid_{di}\neq pid_{dj}$, respectively, observed by the attacker at $RSU_{i}$ and $RSU_{j}$ are unlinkable to the same vehicle from attacker’s viewpoint.

Moreover, $\langle pid_{di},loc_{i}\rangle$ pairs are only known to the sRSUs authorized by $V_{d}$ by means of a re-encryption technique. Another possible attack for an outside attacker is to compromise an sRSU to extract $V_{d}$’s pseudonyms and driving trajectory data $\langle pid_{di},loc_{i}\rangle$ kept by the sRSU on the VANET. As a countermeasure to this threat, in this paper, we just assume that all RSUs are inspected by the TA and compromise of an RSU can be preventable and detectable by means of a security module such as tamper proof device. RSUs would not disclose any information without the authorization of the TA. Nevertheless, if an sRSU is detected as abused, TA can take an action to recover the sRSU and the vehicle can generate new re-encryption keys for sRSUs to protect the changed trajectory data after that.

### 5.2. Performance

**Figure 4:**
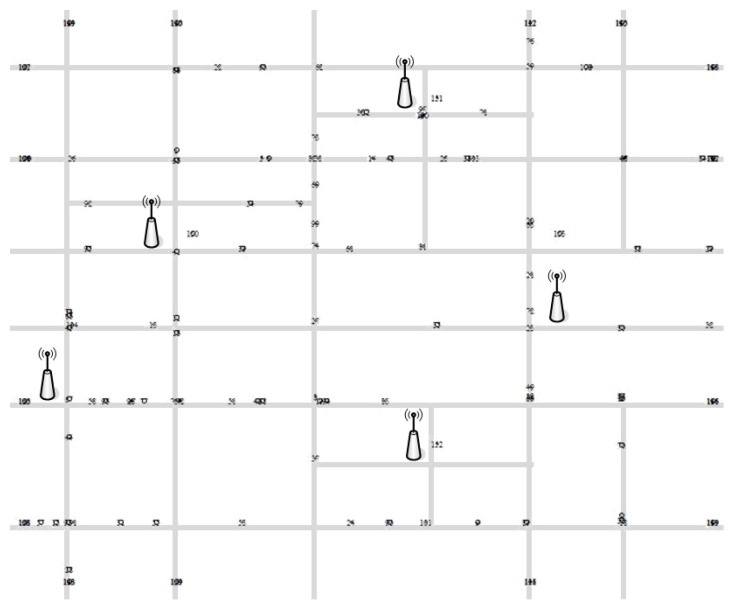
Road configuration for simulation.

We simulated the message delivery on VANETs to evaluate the impact of the proposed security protocol to the performance of message forwarding from RSUs to destination vehicles. We implemented the simulation by using NS-2 and SUMO [30] simulators as considering an urban road environment. Figure 4 and Table 2 show the 4600 m × 3800 m road configuration and simulation settings, respectively.

For our simulation, we applied Manhattan mobility model in which each vehicle moves in horizontal or vertical direction on an urban road and the probability of going straight is 0.5 and taking a left or right is, respectively, 0.25 [31]. We varied the number of vehicles from 30 to 150 moving with 11.1 m/s (40 km/h) to 19.4 m/s (70 km/h) speed on average, and put five contact point RSUs relaying messages to 15 destination vehicles. For message carry-and-forwarding, we adopted the DTN routing protocol of [32] and adjusted message forwarding time to compensate for the delay caused by the authentication process. To measure the authentication overhead of message delivery, we used the benchmark results of pairing-based cryptography library [33] implemented on Intel Quad Core2 2.4 GHz machine by using the supersingular curve $y^{2}=x^{3}+x$ for the group $G_{1}$ with 512-bit base field size and 160-bit group order providing 1024-bit security. Then, we evaluated the performance in terms of message delivery delay and successful delivery ratio on average of 15 destination vehicles for each experiment by varying the number of vehicles and their moving speed.

Let *D* be the total message delay from a contact point RSU to a destination vehicle. The message delay can be estimated as $D=D_{tr}+D_{auth}$ where $D_{tr}$ is the delay for message transmission and $D_{auth}$ is for authentication process resulting from signature generation and verification. Depending on message forwarding method, $D_{auth}$ can be classified as

$$D_{auth}=\left\{\begin{array}{cc} T_{sig}+T_{vrf}, & \mathrm{immediate};\\ 2\left(T_{sig}+T_{vrf}\right)+\sum_{i=1}^{l-1}T_{sig_{i}}, & \mathrm{carry}-\mathrm{forward}. \end{array}\right.$$

where *l* is the number of hops and $T_{sig}$ and $T_{vrf}$ are the times for signature generation and verification evaluated as $T_{sig}$ = 4.65 ms and $T_{vrf}$ = 10.93 ms, respectively. For immediate forwarding, it requires signature generation of the destination vehicle and verification of a contact point RSU before message forwarding. On the other hand, for carry-and-forwarding, authentication of the first carrier vehicle to a contact point RSU and authentication between the last hop vehicle and the destination vehicle are required while each intermediary vehicle only appends its signature to the forwarded message.

**Figure 5:**
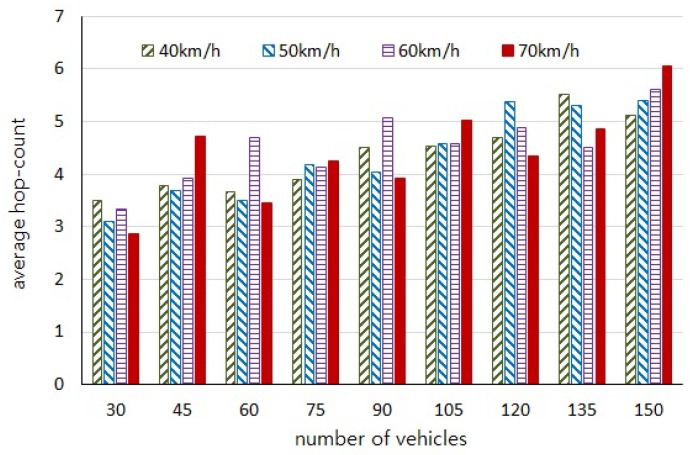
Average number of hops for forwarding messages to the destination vehicles.

**Figure 6:**
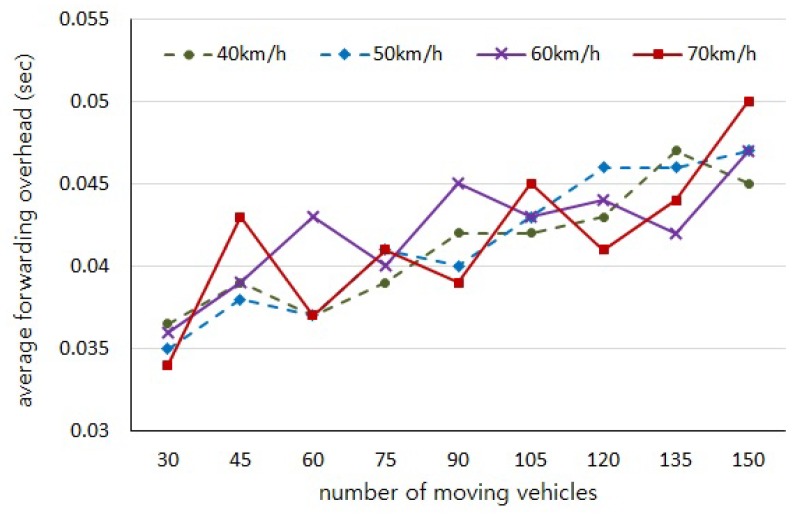
Average forwarding delay ($D_{auth}$) to the number of hops burdened by authentication process.

**Figure 7:**
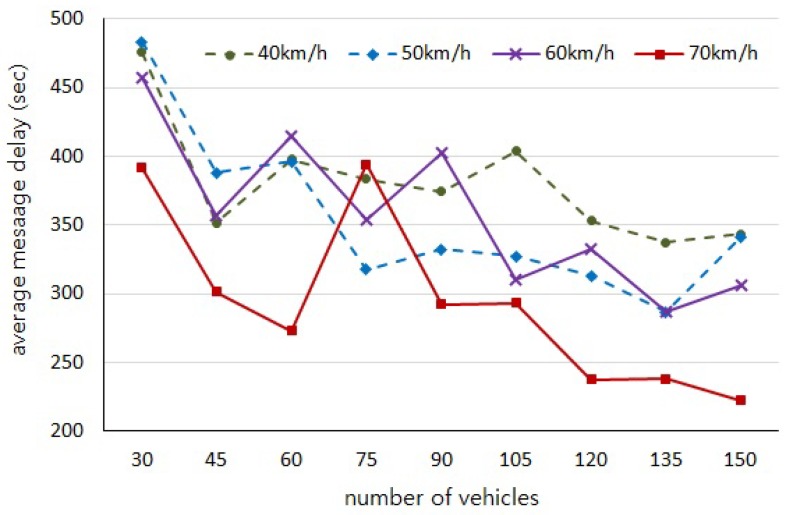
Average message delivery delay (*D*) from contact point RSUs to the destination vehicles.

**Figure 8:**
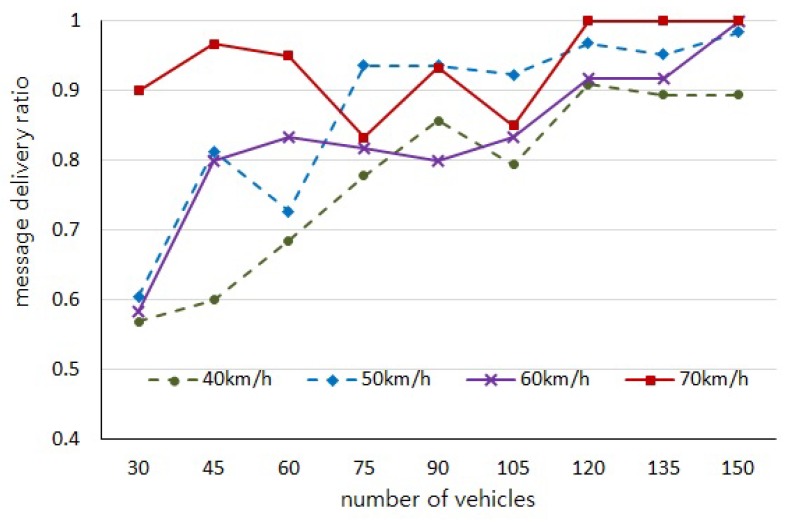
Successful message delivery ratio for the destination vehicles.

With regard to our experiments depending on the number of vehicles and the moving speed, the initial positions of vehicles are randomly generated and the distributions of vehicles on the road in each experiment are not consistent. Thus, those experiments are independent of each other and cause uneven variations in the results of Figure 5, Figure 6, Figure 7 and Figure 8. However, we would like to estimate the performance by observing the trend of changes through the experiment results.

Figure 5 shows the average number of hops, and Figure 6 shows the average authentication delay evaluated by our simulations, respectively. We can observe that the number of intermediary vehicles participating in message forwarding is increased as the more vehicles are distributed on the road. It is obvious that the authentication delay gets longer as the number of hops are increased. However, it should be noted that the more vehicles participate in message forwarding the shorter message delay occurs, as shown in Figure 7, because the carry delay depending on the moving speed of vehicles is much longer than the communication delay. Therefore, the authentication delay is insignificant and has little effect on the total message delay.

We also evaluated the successful message delivery ratio for 500 s of message lifetime and Figure 8 shows the results. As aforementioned, if more vehicles are distributed on the VANET and move with high speed, vehicles can have more chance to meet other vehicles which results in higher possibility of message carrying-and-forwarding as well as shorter message delay. From our simulation results, we can see that almost all of the messages can be successfully delivered to the destination vehicles within 350 s when we put more than 135 vehicles moving with higher than 50 km/h speed.

## 6. Conclusions

Trajectory-based message delivery with the help of roadside units have been considered as an efficient message dissemination method on VANETs under the assumption that message senders know the driving routes of message receiver vehicles. However, from a security viewpoint of location privacy, users of VANETs want to limit sharing of their driving locations to the desired message senders by themselves to prevent the location information from being illegitimately exposed to others. Therefore, in this paper, we propose a secure location sharing system in which vehicles control what roadside units can access vehicles driving locations on their own decision for trajectory-based message delivery services. To effectively share vehicle’s trajectory data with the socialspot roadside units designated by the vehicle on the cloud, we devised a secure trajectory data sharing mechanism by taking advantage of a certificateless proxy re-encryption scheme in which the role of maintaining and distributing encrypted trajectory data can be delegated to a semi-trusted service manager but the access rights to the trajectory data are controlled by vehicles themselves. Therefore, even though vehicles trajectory data are managed by a service manager on the cloud, the trajectory data are hidden from not only unauthorized entities but also the service manager. In addition, whenever vehicles change their preferred driving routes, vehicles can efficiently revoke the access rights of unwanted roadside units to the updated trajectory data just by updating re-encryption keys without involvement of the service manager and roadside units. Consequently, we can design a more flexible and self-controllable secure trajectory data sharing system on VANETs.

## Figures and Tables

**Table 1:** Notations and descriptions.

Notation	Description
$G_{1}$, $G_{2}$	bilinear map groups of a prime order *q*
$e:G_{1}\times G_{1}\to G_{2}$	bilinear map
$g,h\in G_{1}$	generators of $G_{1}$
$s\in Z_{q}^{*}$	master secret key of TA
$g^{s}$	public key of TA corresponding to *s*
$RSU_{i}$	identity of a roadside unit
$sRSU_{j}$	identity of a socialspot RSU
$rsk_{j}$	id-based secret key for an $RSU_{j}$
$pid_{di}$	*i*-th pseudonym of a vehicle $V_{d}$
$vsk_{di}$	id-based private key of a vehicle $V_{d}$ for $pid_{di}$
$sk_{X}$, $pk_{X}$	private and public key of *X* for CL-PRE
$rk_{V_{d}\to sRSU_{j}}$	re-encryption key of $V_{d}$ to $sRSU_{j}$
ts	current timestamp
$Enc_{k}\left(\right)$	symmetric encryption under the key *k*
$Dec_{k}\left(\right)$	symmetric decryption under the key *k*
$MAC_{k}\left(\right)$	message authentication code under the key *k*

**Table 2:** NS-2 simulation parameters.

Simulation Setting	
road dimension	4600 m × 3800 m
# of vehicles	{30, 45, 60, 75, 90, 105, 120, 135, 150}
# of contact point RSUs	5
# of destination vehicles	15
moving speed	{40, 50, 60, 70} km/h
mobility model	Manhattan model
wireless/bandwidth	802.11 p/6 Mbps
radio coverage	250 m
message size	100 KB
message lifetime	500 s
simulation time	2000 s
